# A twin convolutional neural network with hybrid binary optimizer for multimodal breast cancer digital image classification

**DOI:** 10.1038/s41598-024-51329-8

**Published:** 2024-01-06

**Authors:** Olaide N. Oyelade, Eric Aghiomesi Irunokhai, Hui Wang

**Affiliations:** 1 School of Electronics, Electrical Engineering and Computer Science, Queen’s University Belfast, Belfast, BT9 SBN UK; 2 Department of Computer Science, Federal College of Wildlife Management, New-Bussa, Nigeria

**Keywords:** Breast cancer, Computer science, Computational biology and bioinformatics

## Abstract

There is a wide application of deep learning technique to unimodal medical image analysis with significant classification accuracy performance observed. However, real-world diagnosis of some chronic diseases such as breast cancer often require multimodal data streams with different modalities of visual and textual content. Mammography, magnetic resonance imaging (MRI) and image-guided breast biopsy represent a few of multimodal visual streams considered by physicians in isolating cases of breast cancer. Unfortunately, most studies applying deep learning techniques to solving classification problems in digital breast images have often narrowed their study to unimodal samples. This is understood considering the challenging nature of multimodal image abnormality classification where the fusion of high dimension heterogeneous features learned needs to be projected into a common representation space. This paper presents a novel deep learning approach combining a dual/twin convolutional neural network (TwinCNN) framework to address the challenge of breast cancer image classification from multi-modalities. First, modality-based feature learning was achieved by extracting both low and high levels features using the networks embedded with TwinCNN. Secondly, to address the notorious problem of high dimensionality associated with the extracted features, binary optimization method is adapted to effectively eliminate non-discriminant features in the search space. Furthermore, a novel method for feature fusion is applied to computationally leverage the ground-truth and predicted labels for each sample to enable multimodality classification. To evaluate the proposed method, digital mammography images and digital histopathology breast biopsy samples from benchmark datasets namely MIAS and BreakHis respectively. Experimental results obtained showed that the classification accuracy and area under the curve (AUC) for the single modalities yielded 0.755 and 0.861871 for histology, and 0.791 and 0.638 for mammography. Furthermore, the study investigated classification accuracy resulting from the fused feature method, and the result obtained showed that 0.977, 0.913, and 0.667 for histology, mammography, and multimodality respectively. The findings from the study confirmed that multimodal image classification based on combination of image features and predicted label improves performance. In addition, the contribution of the study shows that feature dimensionality reduction based on binary optimizer supports the elimination of non-discriminant features capable of bottle-necking the classifier.

## Introduction

The challenge of addressing increasing cases of breast cancer has motivated widening and intensification of research in the domain. This is necessary considering that fact that breast cancer case count is racing up the ladder as it now currently being rated the second cause of death after cardiovascular diseases^1^. The use of deep learning methods has been widely applied to addressing the problem of early detection of the disease. This approach has demonstrated outstanding performance in reporting impressive classification accuracy and also synthetization of data for supporting the training of the models. However, the use of the deep learning models has often been limited to single modality of breast cancer imaging. Studies which have addressed abnormality classification on single modality have often considered magnetic resonance imaging (MRI)^2^, digital mammography, and ultrasound technology^3^, mammography^4–7^, contrast-enhanced mammography^8^, digital tomosynthesis^9^, sonography^10^, sonoelastography^11,12^, magnetic elastography, diffusion-weighted imaging^13^, magnetic spectroscopy, nuclear medicine^14,15^, image-guided breast biopsy^16–18^, optical imaging^19,20^, and microwave imaging^21^. The unimodal approach to detection of breast cancer disease is limited to using insufficient information in diagnosing physical condition. This has led to improvement in the imaging technology using more advanced methods such as computed tomography (CT), positron emission tomography (PET), single-photon emission computed tomography (SPECT) and even the popular magnetic resonance imaging (MRI). However, it has been reported that the utilization of multimodal methods for information collection through data fusion in diagnosing the disease, provides richer information and separate views so that error resulting from process is minimized^22^. Another study has shown that the combination of mammography and ultrasound modalities have helped to increase the sensitivity of deep learning models by 23%^23^. These further confirms that the use of multimodal method to characterization of abnormalities breast cancer will promote efficient treatment and therapies with increased survival rate and reduced adverse effect^24^.

The need to extract relevant patterns suggesting the detection of the disease has now focused research using deep learning on multimodal data streams. It is assumed that such a multi-stream drawn up pattern can enhance the automation of complex operational processes with capability to improve the diagnosis of the disease. Moreover, it has been observed that the approach multimodal image analysis has traditionally the de-factor technique for zeroing on diagnosis for some severe diseases like cancers^25^. This supports the notion that multimodal deep learning methods reflect the human cognitive use of several modalities features as yardstick to predictions^26^. Single modality which are often annotated with labels for input to deep learning models for detection and classification purpose, suffers from inaccurate and incomplete procedure since the complexity and variability clinical features are lacking^27^. As a result, single modality deep learning models underperforms when compared with deep fusion strategies which leverages on the combination of complex feature representations demonstrating the interactions of different levels of biological processes^28^. Furthermore, the increasing nature of multimodality in biomedical data and the constrained information represented in a single modality are motivations for obtaining sufficient information for disease diagnosis^29^. With this, the unimodal learning is fast becoming obsolete so that the multimodal represents state-of-the-art owning to its capability to improve the robustness of models with the diversity of data^30^, and this has been widely applied to speech recognition, image processing, sentiment analysis and forensic applications. The multimodal approach has the advantage of uniformly analyzing heterogeneous features and fuses them into a common representational space. The fused feature provides classifiers with input representing the contextual nature of the problem domain. This issue of feature fusion has therefore been approached from the perspectives of fusion of multimodal data, fusion deep learning methods, fusion by multimodal transfer learning, fusion by alignment, fusion by multitask learning, and fusion by zero-shot learning^31^. These approaches notwithstanding, the fusion of multimodal sources still suffers conflicts between data sources. Some other challenges of multimodal learning relate to the issue of dimensionality reduction of large features set, confusion between various data sources, and unavailability of multimodal data for extraction of discriminative feature maps.

The challenge of dimensionality reduction of features extracted using deep learning methods has been addressed using optimization methods^4^ for single modality image inputs. However, handling the problem of multimodality with respect to eliminating the bottlenecking effect of large features remains unaddressed. Although textual modality when served as input to machine learning classifiers have benefited from the use of binary optimization methods^32^ in dimensionality reduction on features extracted. Research in the use of visual single modality such as the medical images, usually yields a staggering number of features as output from the convolutional-pooling layers of deep learning. The multimodality will therefore require a scalable and robust approach to managing fused features in a manner described for the textual modality. The use of metaheuristic algorithms as optimization methods remains desirable for addressing this, and which is one of the major issues addressed in this study.

Several studies have investigated the use of deep learning fusion methods on multimodal breast cancer inputs. One reoccurring method is the use of transfer learning to overcome the problem of inadequate heterogeneous data sources for demonstrating multimodality. Also, the use of attention-based multimodal deep learning and bi-modal attention have been used in^33^. Similarly, the use of a weakly supervised clustering-constrained attention multiple instance learning (CLAM) method has been used to overcome the problem of insufficient data in multimodal feature fusion^34^. Multimodal fusion framework (MFF) which relies on a weighted multimodal U-Net (WMM-UNet) have also been proposed with EmbraceNet used to ensemble multimodal features^35^. Another approach to the use of transfer learning in multimodal fusion learning was demonstrated using social engineering optimization with deep transfer learning on photoacoustic multimodal imaging (PAMI)^36^. Another important deep learning technique suitable for aiding multimodal feature learning is the Siamese neural network. Unfortunately, we found that most applications of Siamese networks are in unimodal situations with only a few uses cases nearing multimodal approach. For instance, Siamese network has been used to examine the location of disease and the site of change using medical images from a patient at different time points^37^. Textual modality using a bidirectional gated recurrent unit (BiGRU) Siamese network, and another convolutional neural network based Siamese network was also reported in^38^ for obtaining accurate medical answers. The unimodal Siamese networks described in^37^ and^38^ serves as inspiration and base methods for solving multimodal classification problem described in this study. As a result, we studied other related Siamese networks to observe current gaps in literature with regards to solving feature fusion learning. For instance, triplet Siamese network have been applied to unimodal CT scan images^39^, one-shot Siamese network have solved unimodal classification problem using hyperspectral images^40^, Siamese neural network and k-nearest neighbor (K-NN) model have been used on unimodal brain MRI for neighborhood analysis^41^. Also, the work of^42^ have applied twin neural network in comparing a query versus database samples of optical coherent tomography (OCT) scans for similarity check. The challenge with these methods is the unimodal approach to solving classification problems in medical images whereas multimodal approach presents a more accurate and acceptable classification model. Moreover, we consider that the use of Siamese neural network technique in these studies is under-utilized considering the viability of the technique in measuring similarity distance between learned feature sets. This current gap in recent studies on multimodal medical image classification is the motivation for this study.

Motivated by the existing gaps in the related works, this study is aimed at improving methods for multimodal image fusion in breast cancer detection. In addition, the difficulty of handling very high-dimensional features resulting from multimodalities are addressed in the study. The study therefore seeks to address the deficiency observed in the use of Siamese neural network in in^37,42^ and^38^ which uses visual and textual inputs respectively for solving unimodal classification problems. However, even multimodal feature learning presents another problem associated with dimensionality reduction, effective fusion of features, and computability of the similarity of learned features. In this study, a novel approach to addressing this problem using a single solution of TwinCNN framework is proposed. First, multimodal feature learning is achieved using a tunnel of feature learning represented in the Twin neural network. Secondly, to address the notorious problem of dimensionality reduction in the features resulting from the neural network operation, binary optimization method is adapted to effectively eliminate non-discriminant features with lower pixel value in the search space. This is aimed at ensuring that the similarity computation method is not overwhelmed with non-relevant inputs while also noting that the classifier is not bottlenecked with noisy extracted image features. Furthermore, a novel method for fusing features is proposed by taking consideration of the image label. This approach is completely new and represents state-of-the-art in terms of using Twin neural network for addressing multimodal classification problems in medical image analysis. The following are the contribution of the study:

- Designed a novel TwinCNN architectures named by hsitoCNN and mammoCNN.
- Mechanized the interfacing of binary optimizer to optimize features of the TwinCNN architectures.
- Designed of a novel fusion layer which combines the multimodality probability map distribution.
- Applied data augmentation technique to multimodal data inputs to class-balance the samples.
- Used BEOSA method to a combinatorial and selection problem of 3-class probability map for the multimodal representation.

The remaining part of this paper is organized as follows: a review of related studies with emphasis on current gap in literature is presented in section “Related works”; the methodology of the proposed application of multimodal framework design is presented in section “Proposed method”; In section “Experimentation and multimodal image datasets”, computational resources used, and the dataset applied to the experimentation are discussed. Results obtained from the experiment conducted are comparatively analyzed in section “Results and discussion”. In section “Conclusion”, conclusion on the study is presented, with emphasis made of the possible future works.

## Related works

In this section, we focus on review of recent studies which have applied Siamese CNN architectures to solving unimodal, and some forms multimodal classification problems in medicine and some other domains. Also, a review is provided for studying the trends in the use of deep learning model for solving multimodal classification problems for medical image analysis other than breast cancer. This is necessary because we found very little research effort which have investigated multimodal images relating with breast cancer using deep learning.

### Hybrid and Twin/Siamese CNN architectures

The necessity of using Stochastic Gradient Descent (SGD) algorithm to train fully convolutional Siamese neural network was emphasized in^43^ to solve the problem of online tracking using object detection in video. The aim of this approach was to ensure the weights of the network are well optimized. A closer use of Siamese network similar to solving the multimodal problem was reported in^37^ where the neural network evaluates disease severity at single time points by using two medical imaging domains. Multimodal images based on retinopathy of prematurity (ROP) in retinal photographs and osteoarthritis in knee radiographs were compared to a pool of normal reference images to achieve the disease severity ranking. The study applied the Siamese model on paired images from the same patient as obtained at two different time points to localize the disease and find the site of changes in disease progression. An unrelated use of Siamese network to finding the similarity between online user’s medical question and medical answers have been reported in^38^. Duplet Siamese networks were used consisting of a bidirectional gated recurrent unit (BiGRU) deep learning model, and another convolutional neural network based Siamese network. The first Siamese network was adapted to measure matching similarity for medical interrogation, while the second Siamese network was applied to local information of interrogative sentences with the aim to capture local position invariance. Word vector processing was achieved by the study using Word2Vec method, and an attention mechanism for assigning weights values to keywords in questions. Similarly, a triplet Siamese neural networks which uses few-shot learning algorithms have been investigated in^39^. The study leverages on the benefit of few-shot learning which is capable of effectively learning features from small dataset, to address the problem of detecting COVID-19 CT scan images. In similar work, authors have proposed the use of one-shot single Siamese neural network which was designed with a handcrafted feature generation network that extracts discriminative features from hyperspectral images. The unimodal features extracted was applied to solve classification problem on hyperspectral images to exploit spatial context and spectral bands jointly^40^. Another use of Siamese neural network combined with attention branch loss (ABL) to minimize the challenge of insufficient training dataset^44^. Another use of unimodal approach on Siamese network for the classification of abnormality in brain MRI images is reported in^41^. The Siamese neural network was supported by a k-nearest neighbor (k-NN) model to achieve neighborhood analysis on feature extracted through the neural network while Euclidean and Mahalanobis distances were applied to measure the difference the features as well. Similarly, Siamese neural network was trained on binary diabetic retinopathy fundus image pair information for solving content-based medical image retrieval task^45^. Also, the work of^42^ have applied twin neural network in comparing a query versus database samples of optical coherent tomography (OCT) scans for similarity check.

A novel classification model for breast cancer diagnosis based on a hybridized CNN and an improved optimization algorithm, along with transfer learning, to help radiologists detect abnormalities efficiently was proposed in^46^. The model was divided into four main phases: Data preprocessing and data augmentation, hyperparameters optimization, the learning phase and performance evaluation. Although the proposed improved marine predators algorithm (IMPA-ResNet50) model and achieved high classification performance in breast cancer detection from mammography images, however the limitations of the work is highly spent out as it; the IMPA algorithm success in determining the values of the hyperparameters of the ResNet50 architecture only, and it may not be generalized to other pretrained CNN architecture, secondly the IMPA-ResNet50 was only implemented to classify mammography images. These results are limited to a specific dataset, MIAS dataset, and CBIS-DDSM dataset and may not be generalized to the other dataset. Authors in^47^ worked on a Hybrid Convolutional Neural Network Model Based on different evolution for medical Image classification, the proposed work use the global optimization ability of Differential Evolution algorithm to regulate the structure of CNN to reduce the optimization time of the classification network CNN when solving image classification problems and improves the accuracy of the algorithm classification. However, the work did not consider the impact of different optimizers on CNN network performance. A hybrid deep convolutional neural model for iris image recognition was presented in^48^, it attempts to solve the problem of limited availability of datasets which affects accuracy of the classifiers as it explores the iris recognition problem via a basic convolutional neural network model and hybrid deep learning models. However, the limitation of the work was noted as the performance measures of the proposed methods are limited to the IIT Delhi database and the performance of the network may fail for other iris databases. Likewise, though the convolutional features are more distinct they consume more computation with deep structures and large data samples. Addressing breast cancer classification using deep convolutional neural networks(CNN) was proposed in^49^, total composition of 9,109 breast tumor tissue microscopic images from breast Cancer Histopathological image classification(BreakHis) dataset was used and the system helps to reduce image training process complexity and eliminate the over fitting problem. The model reported 95.4% of average accuracy of the image level and 96.48% accuracy of the patient level for all magnification factors. Authors in^50^ developed data augmented –aided CNN for detection of abnormalities in digital mammography. It was carried out using Floyd server with the dataset from MIAS database, it successfully demonstrated and classified architectural distortion, asymmetric and macro-calcification abnormalities at 90.62% level of accuracy. However, a limitation was discovered when whole images are applied to the proposed architecture as it was found incompatible. In^51^ a CNN architecture for automatic classification of brain tumor into uncropped, cropped and segment region of interest was presented. However, the grading efficiency was not subjected to MR images with different weights and not applicable for larger image dataset though the work reported a performance with an accuracy of 98.93% and sensitivity of 98.18% for the cropped lesions, while the results for the uncropped lesions are 99% accuracy and 98.52% sensitivity and the results for segmented lesion images are 97.62% for accuracy and 97.40% sensitivity.

Application of deep learning and image pre-processing techniques in detecting coronavirus infection was presented in^52^. The study deployed a CNN architecture model that enhanced image preprocessing mechanism able to detect the presence of coronavirus from digital chest X-ray. The outcome of the research revealed that the proposed model achieved an accuracy of 0.1, recall/precision of 0.85, F-measure of 0.9, and specificity of 1.0 However, the designed and deployed architecture incorporated parameters with high demanding memory. The research in^53^ designed an A.I. based breast cancer detection model by combining mammograms and medical health record. The combination of deep learning and machine learning model could detect breast cancer and it demonstrates the advantage of combining mammography images and clinical data. Nevertheless, the study had some itemize limitations; only small dataset was used to train the model, the variability in the clinical factor in each population was different and many women with benign findings were imported into the study. A CNN based algorithm in object detection and semantic segmentation for the medical analysis was developed in^54^. The work provides a great choice for accurate delineation of tumor margin. However, the model training stage needed a large medical image dataset which was not utilized likewise both object detection and segmentation belongs to supervised algorithms which required experienced doctors to label images. The work developed in^55^; a deep learning base detection model for coronavirus using CT and X-ray image data. The system examines the efficiency of CNN, mixture of multiple trained CNNs to automatic identify coronavirus from CT scans and X-ray pictures. Moreover, the model was a theoretical framework which was not subjected nor verified in actual clinical practices. The authors in^56^ proposed a novel wavelet decomposition and transformation CCN with data augmentation for breast cancer detection using digital mammogram. A hybrid algorithm of seam carving and wavelet decomposition to support feature enhancement in the image preprocessing was designed. Microscopic analysis for detecting and confirming cholera and malaria epidemic pathogen using CNN architecture was proposed in^57^. The CNN model achieved classification accuracy of 94%, with 200 Vibrio cholera images and 200 Plasmodium falciparum images for training dataset and 80 images for testing data which can provide significant help epidemic crisis, nevertheless work is practically limited to small datasets. The authors in^58^ proposed an automatic classification of medical image modality and anatomical location using CCN. Four groups of images were created: CT (computed tomography) of abdomen, CT of brain, MRI of brain and MRI of spine. The images were converted in JPEG and the proposed CNN architecture classified the medical images based on anatomic location and modality. The performance metrics on the classification accuracy in both validation and test set (> 99.5%) and F1 score (> 99%) in both diseased and normal image category of dataset. However, the study had some limitations; the images were limited to JPEC image format for the proposed CNN architecture and there was no preprocessing medium for the images. In addition, only a small dataset was used while just two MRI classes were considered.

### Multi-modal CNN architectures optimized using metaheuristic algorithms

Authors in^59^ have proposed the use of deep learning and body map to classify wounds and their location in the body using multimodal approach. The study combined images with wounds and their corresponding images with location were applied to their model. The fusion of the features extracted from the two modalities was achieved so that both image-based and location-based features were supplied to the classifier. In another work, authors approach the use of multimodality with deep learning with the aim of solving image segmentation problems. The study focused on using self-attention mechanism on all modalities of inputs so that different anomalies features are extracted. The self-attention mechanism combined a deep learning encoder-decoder for the segmentation task^25^. The need to eliminate noise and distortion in data stream associated with electrocardiography (ECG) has been addressed using multimodal deep learning method which combines other data streams for improved diagnosis^30^. The fusion of data streams from several 3D neuroimaging into a pattern representing an informative latent embedding has been investigated. The study applied a deep learning architecture which was designed from the generative model’s approach which allows for separation of convolutional blocks in modular approach. The aim is to draw out patterns of phenotypic from brain images to support using biomarkers for charting spatio-temporal trajectories^27^. The problem of inaccuracy in classification of brain tumor is being addressed using multimodal deep learning method. The combination of firefly Optimization algorithm and convolutional neural network which uses a modified fully connected layer was used to address this problem. Features extracted from different modalities were fused so that only the lower-level, middle-level, and higher-level image contents are extracted^29^. The combination of extreme learning machines and convolutional neural network have been proposed for feature extraction and fusion on multimodal images to support the classification accuracy and localization of medical images^60^. Another approach to multimodality is the consideration of multicolor imaging for the purpose of extracting features which reveal sufficient symptoms to arrive at the detection of diseases. Using deep learning networks, a study has shown that diabetic cases can be detected from fundus images when multimodal information bottleneck network (MMIB-Net) was applied to classify features extracted from the multicolored input source^61^.

The design and implementation of an image segmentation system based on deep convolutional neural networks to contour the lesions of soft tissue sarcomas using multimodal images, including those from magnetic resonance imaging, computed tomography, and positron emission tomography was proposed in^62^. The network trained with multimodal images shows superior performance compared to networks trained with single-modal images. Nevertheless, the framework was only tested on a single dataset using one set of simple network structures. Authors in^63^ presented Breast cancer histopathological image classification using convolutional neural networks, used high-resolution histopathological images, however for training, only small patches of the images are used. Early diagnosis of oral cancers using three-dimensional convolutional neural networks was proposed in^64^. The early diagnosis of oral cancers used 3DCNN, and then constructs a deep 2DCNN and 3DCNN, but the work was limited to a small amount of existing sample data. Authors in^65^ proposed a deep learning approach based on a Convolutional Neural Network (CNN) model for multi-class breast cancer classification was presented, the study classify the breast tumors in non-just benign or malignant and able to predict the subclass of the tumors like Fibroadenoma, Lobular carcinoma but the work only made used of smaller dataset. Breast cancer histopathological image classification using a hybrid deep neural network was presented in^66^ but suffers lack of pre-processing data.

The fusion learning using breast cancer image modalities have received considerable research attention. Authors in^33^ have proposed the use of attention-based multimodal deep learning model by first extracting the features of different modalities using sigmoid gated attention convolutional neural network. Thereafter, bi-modal attention mechanism is applied to further identify subtle patterns or abnormalities to obtain insightful patterns which can support the prognosis of the disease. In another study, authors investigated the performance of a weakly supervised clustering-constrained attention multiple instance learning (CLAM) as combined with ResNet and EfficientNet-B0 architectures. The neural architectures were first trained on ImageNet dataset before being exposed to extract features from multi-view forms of mammography. The study showed that the attentional maps concentrated the workflow on relevant fused features, and with some measure of explainability which can eliminate erroneous predictions^34^. A multimodal fusion framework (MFF) which relies on a weighted multimodal U-Net (WMM-UNet) model has been proposed for segmenting lesions. The framework combines a decision network and an integrated feature network to learn multimodal features from B-mode and strain elastography mode when ultrasound images are supplied to multiple CNNs. The study uses multimodal EmbraceNet to fuse the features learnt using the CNN models^35^. A two-level machine learning technique had been applied to separately extract unimodal information from two different images sources to classify and then predict breast cancer. The study combines ultrasound features with clinical and demographic information to achieve the model^67^. In^36^, the need to use social engineering optimization and transfer learning on enhancing photoacoustic multimodal imaging (PAMI) fusion was proposed. The authors noted that using social engineering optimization with deep transfer learning can improve the process. ResNet-18 and a lightweight LEDNet were first applied to feature extraction and segmentation tasks, while bilateral filtering (BF) was used to preprocess the image inputs. Furthermore, social engineering optimization was utilized on recurrent neural network (RNN) model to aid class labeling of the biomedical images. The multimodal nature of microscopic imaging which combines bright-field, auto-fluorescence and orthogonal polarization images presents a way for extracting, fusing, and analyzing the multimodal features. The use of deep learning method has been proposed in^68^ to obtain rich information of tissue morphology, content, and structure of collagen in tissue slices from the fused features set. On the other hand, the multimodality of MR images has also been investigated when using the extracted features with clinical information to predict pathology complete response (pCR) to neoadjuvant chemotherapy (NAC). The approach also uses a deep learning model for the fusion of the multimodal features from clinical information, T1-weighted subtraction images, and T2-weighted images^69^. In Table 1, we provide a summary of the review considered in this section.

**Table 1:** Comparison of the related work showing the pros and cons.

Author and year	Pros	Cons
^43^, 2016	Ensure the weights of the network is well optimized	
^62^, 2016	Breast cancer histopathological image classification	Small patches of the images are used
^56^, 2018	Detecting and confirming cholera and malaria epidemic pathogen	Practically limited to small datasets
^64^, 2018	Multi-class breast cancer classification able to predict the subclass of the tumors	Ssmaller dataset was used
^61^, 2019	Improved performance on image segementation	Framework was only tested on a single dataset using one set of simple network structures
^63^, 2019	Diagnosis of oral cancer	Limited to small amount of existing sample data
^24^, 2020	Self-attention mechanism on all modalities of inputs so that different anomalies features are extracted	
^37^, 2020	Evaluates disease severity at single time and find the site of changes in disease progression	
^46^, 2020	Regulate the structure of CNN to reduce the optimization time and improves the accuracy of the algorithm classification	Account the impact of different optimizers on CNN network performance not considered
^65^, 2020	Breast cancer histopathological image classification	Lack of pre-processing data
^41^, 2021	Classification of abnormality in brain MRI images	
^48^, 2021	Reduce image training process complexity and eliminate the over fitting problem	
^50^, 2021	Automatic classification of brain tumor into uncropped, cropped and segment region	Not applicable for larger image dataset
^51^, 2021	Enhanced image preprocessing mechanism able to detect the presence of coronavirus from digital chest X-ray	Deployed architecture incorporated parameters with high demanding memory
^53^, 2021	Great choice for accurate delineation of tumor margin	Both object detection and segmentation belongs to supervised algorithms which required experienced doctors to label images
^54^, 2021	Detection model for coronavirus using CT and X-ray image data	The model was a theoretical framework which was not subjected nor verified in actual clinical practices
^57^, 2021	Cclassified the medical images based on anatomic location and modality	Images were limited to JPEG, no preprocessing medium for images and it was subjected to small dataset
^68^, 2021	Clinical information to predict pathology complete response (pCR) to neoadjuvant chemotherapy (NAC)	
^39^, 2022	Leverages on the benefit of few-shot learning, to address the problem of detecting COVID-19 CT scan images	
^59^, 2022	Multicolor imaging for the purpose of extracting features which reveals sufficient symptoms to arrive at the detection of diseases	
^40^, 2022	Solved classification problem on hyperspectral images to exploit spatial context and spectral bands jointly	
^44^, 2022	Minimize the challenge of insufficient training dataset	
^46^,2022	High classification performance in breast cancer detection from mammography images	May not be generalized to other pretrained CNN architecture and limited to a specific dataset
^47^, 2022	Iris image recognition	The performance measures of the proposed methods are limited to the IIT Delhi database and the performance of the network may fail for other iris databases
^49^, 2022	Demonstrated and classified architectural distortion, assymetric and macro-calcification abnormalities	Proposed architecture not compatible to whole image
^58^, 2022	Wounds and their location in the body using multimodal approach	
^28^, 2023	Feature extraction and fusion on multimodal images to support the classification accuracy and localization of medical images	
^29^, 2023	Eliminate noise and distortion in data stream associated with electrocardiography	
^33^, 2023	Eliminate erroneous predictions	
^52^, 2023	Demonstrates the advantage of combining mammography images and clinical data	Only small dataset was used to train the model

In the nest section, a detailed design and discussion on the methodology applied to our proposed study is presented. This explains the difference between the summary of findings in the current state-of-the-art and what is proposed by the study as a means of closing the existing gap in literature.

## Proposed method

The methodology describing the design of the proposed multimodal CNN framework is discussed in this section. Here, the complete overview of the framework is presented with every component integrated in a manner as to describe the flow of data from input to output. Furthermore, each integral part of the framework is isolated for an elaborate design and discussion. First, the binary optimization, namely the BEOSA method, is presented showing the algorithmic design and optimization process of the approach. Secondly, the layout of TwinCNN architectures is modeled for understanding of how features are being extracted on the multimodal inputs using convolutional layers. In addition to this, we show how the BEOSA method is applied to optimize the features extracted. Thirdly, the novel probability map fusion layer is designed and discussed. The following subsections address these three major components in addition to the overview layout of the approach.

Technically speaking, the proposed framework adapts CNN architectures to a combinatorial problem of learning abnormalities features in breast digital images. The modalities of digital images considered are the histology and mammography samples based on their high detection rates of all categories of abnormalities. To ensure that the curse of dimensionality does not interfere in the feature fusion leading to classification, a novel approach using binary optimization algorithm was applied to address this common problem. Furthermore, a novel method to fusing multimodalities images based on features and predicted label, is also described.

### The multimodal TwinCNN framework

**Figure 1:**
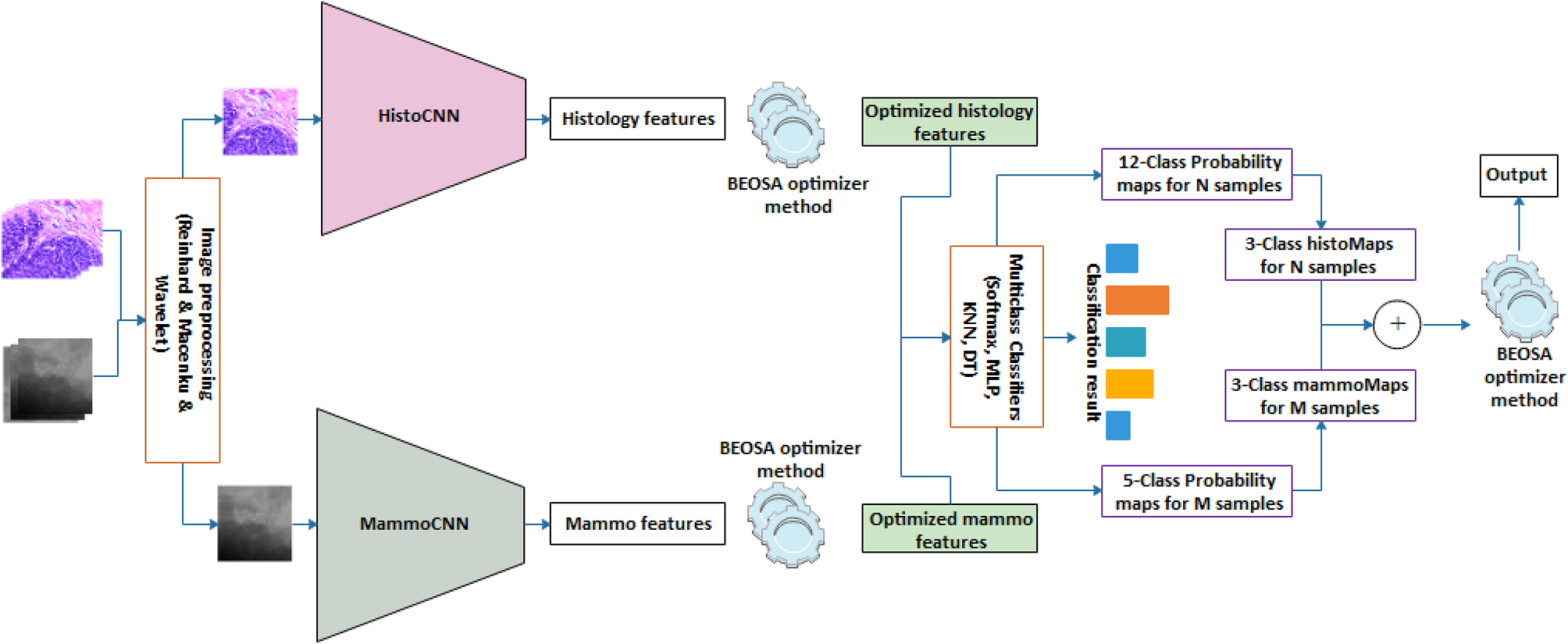
The proposed multimodal TwinCNN framework with BEOSA optimization method for improved classification and characterization of breast cancer abnormalities in digital images.

The framework demonstrating the adaptability of the use of scalable multimodal networks to addressing multi-media sources for breast cancer diagnosis is considered in this subsection. In Fig. 1, the pipeline effect of the framework is outlined with each integral parts showing how the flow branches out to achieve the overall aim of solving classification problem. The figure has nine (9) components which are logically integrated starting from the input right through to the output of the framework these components are: the layered image preprocessing techniques; the CNN networks for feature extraction comprising of the histoCNN and mammoCNN; the feature buffer purposed for storing features drawn from the convolutional layers; the BEOSA method applied for optimization of the features extracted; the buffer for keeping the optimized features; a classifier applied to first classify the optimized features at the level of single-modality; a mapping mechanism for re-representation of the probability distribution of the single-modality to a unified multi-modality aware probability distribution; a probability map fusion layer; and lastly the use of BOESA method for a second level of optimization process.

TwinCNN can extract multimodal features sets. However, this feature representation is high dimensional and contains both discriminant and non-discriminant features which might bottle-neck the performance of the classifier. As result, the features are formalized into a search space so that the BOESA algorithm iteratively optimizes this search space by evaluating and computing an optimal combination of features which yields good classification accuracy. This evaluation is achieved using Softmax, multi-layer-perceptron, KNN and decision tree classifiers (as shown in Fig. 1). The optimized feature set represents a reduction in dimensionality, which then gets supplied as input to the classifier of TwinCNN.

The stack of image preprocessing methods applied to the inputs to the framework includes the contrast-limited adaptive histogram equalization (CLAHE), wavelet decomposition, and Reinhard^70^ methods. The CLAHE and wavelet decomposition methods were applied to the mammography datasets while the Reinhard method was applied to the histopathology images. The use of the CLAHE method on the samples is to ensure that the contrast of the images is enhanced to improve performance. Moreover, this also supports the attainment of samples with high quality to enable the process of image extraction. The wavelet decomposition method as applied to the samples allows for compression of the pixel representation of images so that non-discriminant features are excluded leaving significantly discriminatory features to pass through the convolutional layers. The use of the Reinhard method on the histopathology samples is to support the image normalization process to position the samples suitably for use. In addition to this, the study applied image enhancement and noise removal methods to the histopathology samples to complete the preprocessing phase.

Once the input is preprocessed, the framework pipes the inputs to their respective feature extraction and buffering phase. This ready the features for application of binary optimization method which serves to blind-out features that are not computed to have little relevance to the classification process. Blind-out features are eliminated leaving the relevant for to be fed to the classifier, while the probability distribution of the batch samples is passed as input to the fusion layer. In the following subsections, the approach of the fusion layers and the BEOSA method on this layer and the CNN feature extraction phase, are discussed.

### BEOSA method

The binary optimizer is popular with use in the feature selection on binary classification problem. Binary Ebola optimization search algorithm (BEOSA) is one of recent state-of-the-art methods^32,71^ derived from the continuous metaheuristic method namely Ebola optimization search algorithm (EOSA)^4,72^. In this subsection, a brief discussion on the optimization process of the BEOSA is presented, with emphasis on the use of this method to address the optimization of features extracted during the convolutional operations.

The binary representational approach to the search space of BEOSA requires that only 0 s|1 s are expected in the dimension of an individual so that the entire population is represented by Eq. (1). Where $p$ is the population size, $ind_{i}$ represents an arbitrary individual in the population $S$, and $dim$ is the dimension of each $ind_{i}$,

1 $$S = \{ ind_{i} | 0 \le i \ge p,\;\;\; ind_{i} = \mathop \sum \limits_{j = 0}^{dim} ind_{i, j} = 1$$

An optimized state of $S$ is achieved after a reasonable number of iterations on it using the BEOSA method which applies Eq. (2) to the process search for best solution in an explorative and locally traversed manner. The use of the $rand$, $\Delta$, and $best$ notations represent randomly generated uniform distribution satisfying $[-1, 1]$, a scaler value for change factor of an individual, and the current best solution in the population respectively.

2 $$ind_{i}^{new} = \Delta *e^{rand} {\text{cos}}\left( {2\pi rand} \right)* (ind_{i} - best)$$

BEOSA optimizer applies S-function and V-function styles as transfer function to transform and smoothen the composition of $ind_{i}$ across its dimension. Note that this smoothening maintains the binary nature of the search space by using the approach described in Eq. (3). Here each item in the dimension of $ind_{i}$ is traversed and new values a computed a random float number $r$ satisfies some condition say $r > S\left( {ind_{i}^{k} } \right)$ or $r > T\left( {ind_{i}^{k} } \right)$, where $ind_{i}^{k}$ is an item along the dimension of $ind_{i}$, $k$ is $0, 1, \ldots dim$.

3 $$ind_{i}^{k} = \left\{ {\begin{array}{ll} 1 &\quad {r > S\left( {ind_{i}^{k} } \right) \left| { r} \right\rangle T\left( {ind_{i}^{k} } \right)} \\ 0&\quad {otherwise} \\ \end{array} } \right.$$

The binary optimizer described is adapted to solve two different combinatorial and selection problems. The first is the optimization of the features extracted by the convolutional layers, and the second is the combinatorial problem of probability distribution at the fusion layer. The algorithmic representation describing the flow of procedure for the BEOSA method is outlined in Algorithm 1. The algorithm demonstrates how the binary optimizer branches into to solving either of the problems depending on the setting of a variable Boolean $is\_feature\_optimized$, this is in addition to three other variables namely the maxIter, srate, and lrate. Output from the algorithm is expected to be the classification result of the optimized probability map distribution.
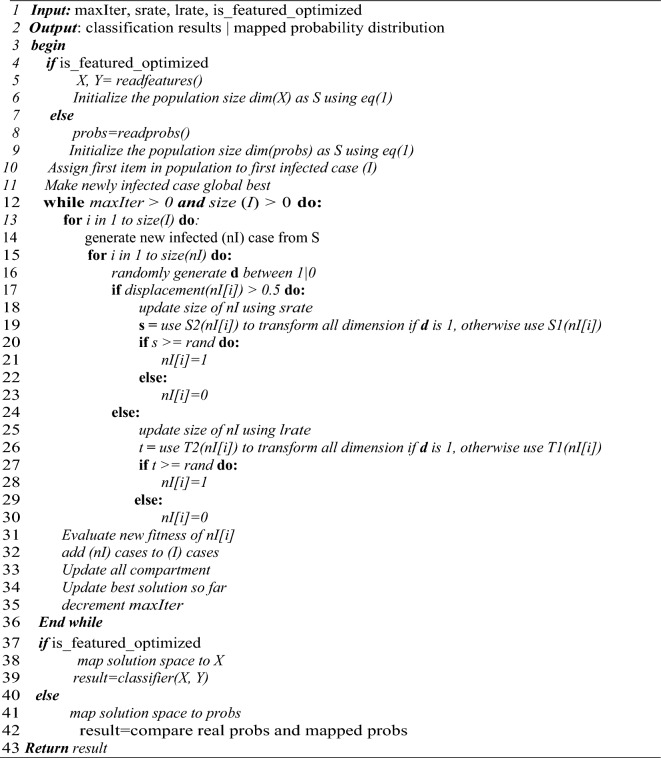


Lines 4–9 of the algorithm shows how the checking for the solution set and space will be configured depending on if the optimization problem is on extracted features or on probability distribution. This is followed by the identification of the current best solution after the fitness of each individual in the solution space must have been computed as seen on Lines10-11. Between Lines 12–36, the iterative training of the binary optimizer is shown where Lines 17–24 illustrates how the S-style-function is being applied, and 25–30 demonstrates V-style-function usage, and at the same time showing exploration and intensification respectively. Variable updates are computed and outlined on Lines 31–36. Between Lines 37–39, the condition for classification of the optimized feature is checked and executed while on Lines 40–42, the optimized combinatorial solution to distribution of probability maps is output. Finally, the result for the algorithm is returned on Line 43. In the following two subsections, we show how each of the concepts described therein apply Algorithm 1.

### TwinCNN architectures

**Figure 2:**
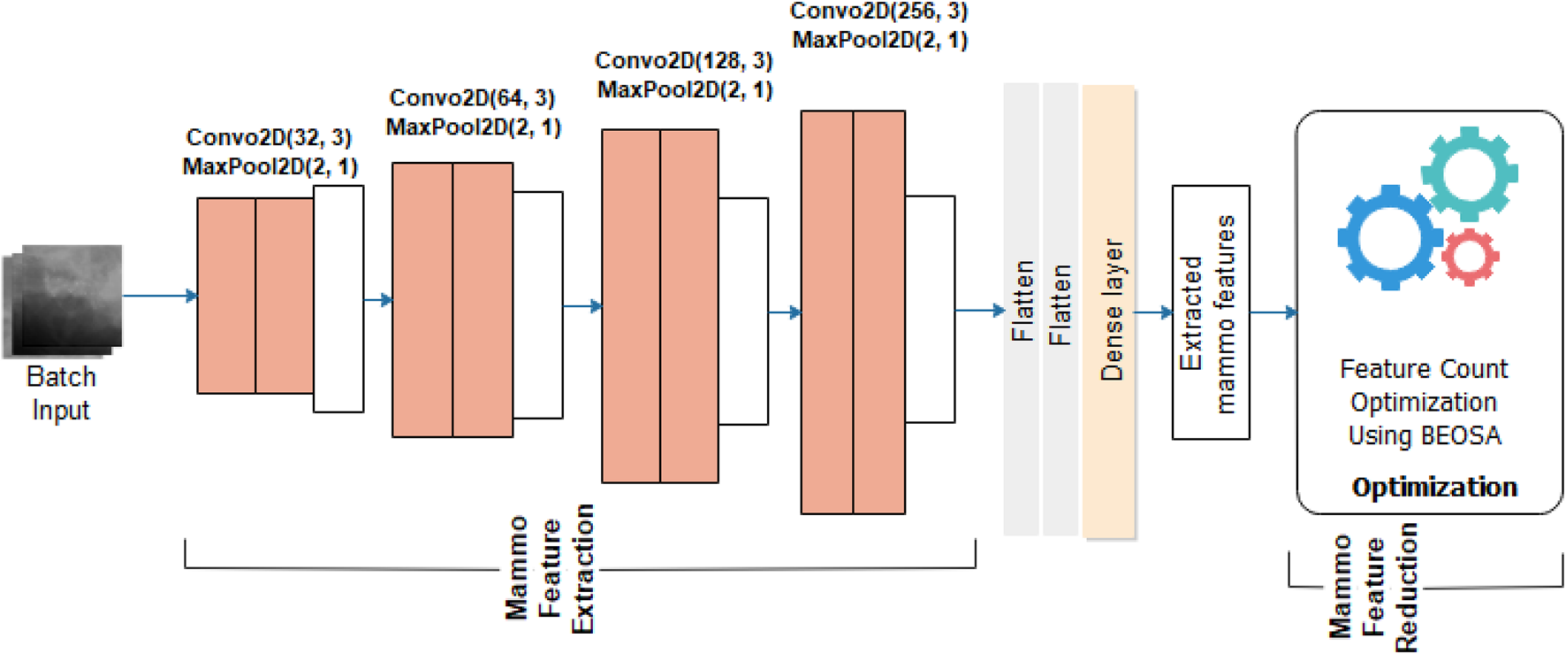
MammoCNN architecture for the feature extraction and feature optimization using digital mammography.

The TwinCNN network used for the feature extraction phase of the design is aimed at two modal features sets. However, the design of the framework allows for scaling up the number of CNN architectures for feature extraction tasks. In this study, abnormality features apply for the classification problem is focused on the histology and mammography samples as computed from digital histopathology and digital mammography images. In Fig. 2, the mammoCNN architecture is illustrated with four (4) blocks of convolutional-pooling operations consisting of two convolutional layers and as appended with a max-pooling layer in each block. Filter size consistently across all blocks remained at 3 × 3 while the filter count follows the order of ${2}^{n}$ where $n=5, 6, 7, 8$. These five blocks of convolutional-pooling operations are followed by two flatten layers and a dense layer having dropout layer at the rate of 0.5.

**Figure 3:**
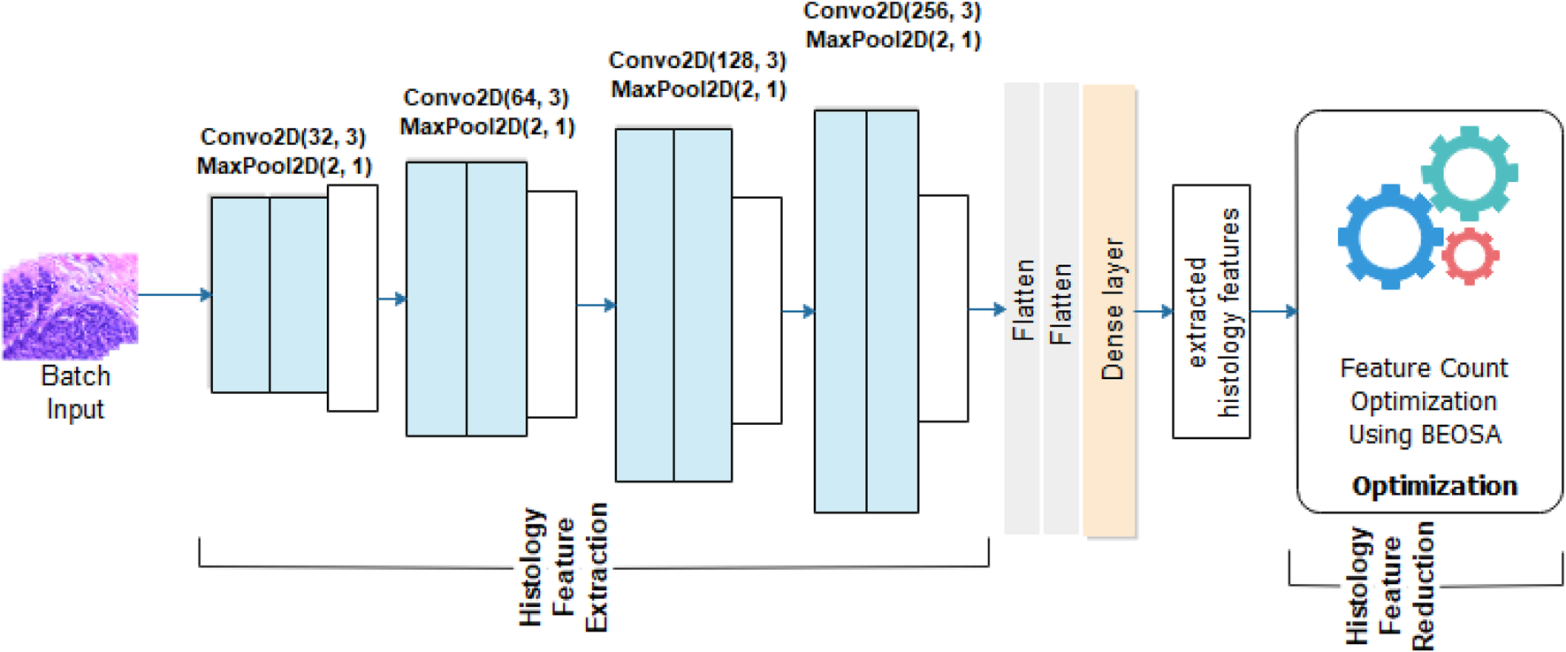
HistoCNN architecture for the feature extraction and feature optimization using digital mammography.

Like the twin network, the histoCNN architecture which is illustrated by Fig. 3 assumes the same five convolutional-pooling operations. Unlike mammoCNN which allows input with 1 channel, the histoCNN accept input having 3-channels. We note that configuration of each block of the convolutional-pooling layer derives its number of filters count like what is obtained for mammoCNN which is ${2}^{n}$ where $n=5, 6, 7, 8$. This implies that the filter count of 32, 64, 128, and 256 were applied to convolutional operations in layers 1, 2, 3, and 4 respectively. Meanwhile, in the case of the histoCNN and mammoCNN, a uniform kernel size of 3 × 3 was implemented in all convolutional layers. The two composing neural architectures of the TwinCNN have their convolutional layers laced with a max-pooling operation with a kernel size of 2 × 2 and stride of 1. Meanwhile, stride size of 1 was applied to every convolutional operation occurring in histoCNN and mammoCNN with input sizes of 224 × 224 and 299 × 299 respectively. This block of convolutional-pooling operations is applied for feature extraction in a multi-level approach as typical of CNN models. This therefore implies that the convolutional-pooling block in mammoCNN is expected to detect discriminant high-level and low-level mammography features through the pipeline. In the same way, histoCNN will extract histopathology features consistent with breast cancer abnormalities in with of the 3-channel modality through the pipeline of convolutional-pooling operations.

Appended to both mammoCNN and histoCNN are feature optimization functionality which turns over the extracted mammography features and histology features respectively to the BEOSA optimizer. The binary optimizer is expected to apply its operations on the feature load in a manner as to return an optimized version of the solution space. The feature load is represented in Eq. (4) where a row represents the feature extracted for an image sample while each column is an indication of pixel representation of the feature contained in it, and where $i = 1, 2, \ldots , N$. Equation (5) shows the fitness function applied by the binary optimizer to check the relevance of each feature as represented in the column of each instance. Where $clf$ is the KNN classifier used, and $\left| F \right|$ is an absolute representation of feature counts obtained from an individual $ind_{i}$.

4 $$img\left[ i \right] = \left[ {\begin{array}{*{20}c} {feature_{1,1} , feature_{1,2} } & \cdots & {feature_{1,dim} } \\ \vdots & \ddots & \vdots \\ {feature_{n,1} , feature_{n,2} } & \cdots & {feature_{n,d} } \\ \end{array} } \right]$$

5 $$fit = 0.99*(1 - clf\left( {img\left[ {:1^{{ind_{i} }} } \right]} \right) + \left( {\left( {1 - 0.99} \right) \frac{\left| F \right|}{{dim}}} \right)$$

It is desirable that the effect of the optimization on $S$ will output something like what is seen in Eq. (6). Features optimized to 0 in the solution space by the binary optimizer are blinded out and not passed onto the pool of optimized feature sets.

6 $$S = \left[ {\begin{array}{*{20}c} {1_{1,1} , 1_{1,2} } & \cdots & {0_{1,dim} } \\ \vdots & \ddots & \vdots \\ {0_{n,1} , 1_{n,2} } & \cdots & {1_{n,d} } \\ \end{array} } \right]$$

The optimized features from mammoCNN and histoCNN are then passed on for a complete classification task in a multi-class classification problem using the softmax classifier shown in Eq. (7).

8 $$\sigma \left( {\overrightarrow {img} } \right)_{i} = \frac{{e^{{\overrightarrow {img}_{i} }} }}{{\mathop \sum \nolimits_{j = 1}^{n} e^{{\overrightarrow {img}_{j} }} }}$$

The outcome of the classification is a probability distribution according to class-labels of digital mammography and histopathology. The distribution is passed on to the fusion layer described in the next subsection.

### Probability map fusion with BOESA

**Figure 4:**
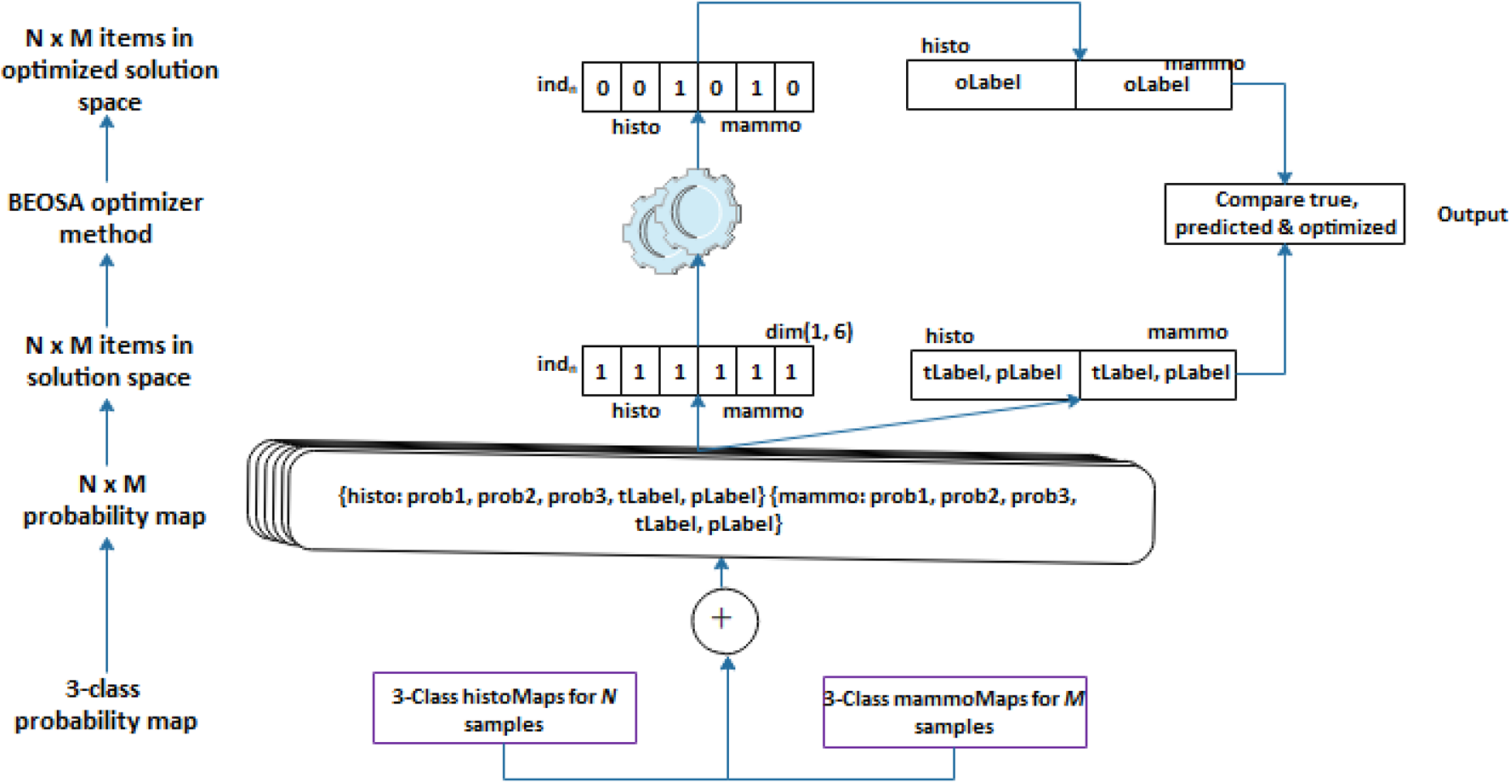
Representation of the fusion layer for the histoCNN and mammoCNN population space and the optimized solution space.

Traditional fusion of CNN architectures often merges the extracted features or combines the neural network layers. In this study, we demonstrate a new approach for combining outcome from two CNN architectures as described in this subsection. In Fig. 4, we show a hierarchical flow of concept and data leading to the composition of the search space and the application of the BEOSA method to optimize the binary search space in a unique way. Using a bottom-up method, the multi-class probability distribution generated from the classification of the optimized feature sets from mammoCNN and histoCNN are combined into a stack of set of probabilities. Note that the class-label for histoCNN follows a five-class-label distribution while that of the histoCNN follows 12-class-label with both neural network outputting $M$ and $N$ samples respectively. The multimodal class-labels are scaled down and uniformed into 3-class-label to allow for the fusion layer work with it by concatenating each item in the 3-class-label representation in histoCNN and then concatenate with an item in the mammoCNN with the same abnormality. This concatenation operation includes the real and predicted label for the two modalities to form $N x M$ items probability map distribution. In the next upper level, the search space is configured in a binary manner for $N x M$ items to constitute the solution space.

The representation of each item in the solution space follows according to Eq. (8), where the first item in the tuple is the positional index of the individual, followed by a 3-element list defining the composition of the individual. The 3-element list has the binary positional representation for the individual, the fitness as computed, and the corresponding 3-class-label for true and prediction of the two modalities.

8 $$ind_{i} = \left. {\left. {\left( {index, \left[ { pos, fit, } \right[p1,p2,p3,p4,p5,p6} \right]} \right]} \right)$$

At the initial stage preceding the optimization cycle, the positional representation of an individual is denoted by Eq. (9), while the computation of the fitness function when the BEOSA method is applied for the optimization process follows Eq. (10).

9 $$pos = \left[ {1,1,1,1,1,1} \right]$$

where $s=3$ and $t=3$ is the maximum number of probability maps in the two modalities, since the varying multi-class labels in the two modalities have been mapped into a 3-class-label. The notations ${p}_{i}$ and ${p}_{j}$ represents corresponding probability values in histoCNN and mammoCNN.

10 $$fit = 2 - \left( {\mathop \sum \limits_{i = 0}^{s} p_{i} + \mathop \sum \limits_{j = s}^{t} p_{j} } \right) \forall i,j pos_{i} ,pos_{j} \ne 0$$

The binary optimizer operates on the solution space to derive a new solution which transforms the default composition of the $pos$ into an optimal representation which allows for best selection and combination of probability maps. This combination leads to computing the projected classification which considers the abnormality in both modalities. In the next section, we present the system configuration and the datasets applied for the experimentation phase of this study.

## Experimentation and multimodal image datasets

In this section, we provide details on the machine configuration which was set up for implementing the proposed system. Meanwhile, the parameter settings for the binary optimizer and those for the histoCNN and mammoCNN are also listed to support reproducibility of the experimentation described in the study.

### Computational setup for experimentation

The implementation of the framework and algorithmic process described in the last section was achieved using Python. In addition, some python-based libraries were also used including deep learning libraries tensorflow and keras, numpy, and matplotlib. The computational setup which allows for the experimentation was achieved using the Google collaboration (Google Colab) platform. This platform provided us with 12 GB memory and disk size of 100 GB, both connected with Python 3 Google computer engine backend with a graphical processing unit (GPU). Further experimentation was then carried out using the Google cloud compute engine with an instance spawned using the following configurations: machine type of n1-highmem-8 on the Intel Broadwell central processing unit (CPU) platform of Intel Broadwell, boot disk of 200 GB, 8 vCPUs, and 50 GB memory. Furthermore, the multimodal model was trained on a system with the following configuration: Intel Silver Xeon 4210, 10 CPU scores, 20 threads, 256 GB RAM, 4 TB HDD, 960 GB SSD, Tesla V100 32 GB * 2 GPU.

Implementation of the histoCNN and mammoCNN were based on the combination of python libraries which includes the TensorFlow, Keras, NumPy, Matplotlib, and the Sklearn where classifiers such as the KNeighborsClassifier, MLPClassifier, and DecisionTreeClassifier were utilized. For the BEOSA algorithm, several of those libraries such as the Numpy, Scipy.stats.expon, Pandas, and basic libraries such as the math and random. These form the major libraries used for the implementation of the TwinCNN framework, though other very popular libraries were also used where necessary.

### Parameter settings

In Table 2 is a listing of parameter settings for the experimentation with histoCNN, mammoCNN, and the BEOSA method, as applied for the optimization process. The table provides a description for every parameter, and a corresponding value applied for that parameter. Notations were adopted for representation of each parameter as used in the framework.

**Table 2:** Parameter settings for the histoCNN, mammoCNN, and BEOSA method.

Method	Parameter	Value	Description
histoCNN and mammoCNN	$\partial , \alpha , {\beta }_{1},$ and ${\beta }_{2}$	1e−06, Adam, 0.5 and 0.999 respectively	The learning rate, optimizer algorithm, beta1 and beta2 respectively
	$\varepsilon , \tau ,$ and $\varphi$	1e−08, 0.0002, and 32 respectively	Epsilon, L2 regularizer rate, and batch size respectively
	${w}_{h} \times {h}_{h}$ and ${w}_{m} \times {h}_{m}$	224 × 224, and 299 × 299 for histoCNN and mammoCNN respectively	Image input sizes for histoCNN and mammoCNN
	${t}_{s}$,${e}_{s}$ and ${p}_{s}$	0.75, 0.15, and 0.10	Train split, evaluation split and test split for the datasets
BEOSA	N	0.1	Recruitment rate
	p1, p2, p3 and p4	0.1, 0.1, 0.1, and 0.1	Contact rate of infected individuals, of the host, with the dead, and with the recovered individuals

In the following subsection, detailed information on the multimodal datasets used for the experimentation are described.

### The multimodal image dataset

The medical image datasets combined includes those from the histology and mammography modalities. The histology samples were sourced from two major publicly accessible databases namely the BreakHis^73,74^ dataset, and the BACH^75^. The combination of samples from these two datasets provided us with a rich and enough image samples to train and evaluate the histoCNN model. The training of the mammoCNN was achieved using hybrid datasets collected from the publicly accessible database named Mammographic Image Analysis Society (MIAS)^76^ and Curated Breast Imaging Subset (CBIS) of the Digital Database for Screening Mammography (DDSM + CBIS)^77^, which we obtained the samples in numpy representation. For the histology, a total of 7441 samples were applied with adenosis (A) having 456 samples, (B) having 100 samples, malignant carcinoma (DC) having 2749 samples, fibroadenoma (F) having 1127 samples, in situ carcinoma (IS) having 100 samples, malignant invasive carcinoma (IV) having 100 samples, malignant lobular carcinoma (LC) having 426 samples, malignant mucinous carcinoma (MC) having 495 samples, (N) having 96 samples, malignant papillary carcinoma (PC) having 348 samples, phyllodes tumor (PT) having 469 samples, and tubular adenona (TA) having 630 samples. For the MIAS samples, a total of 3104 samples were sourced with the class labels distributed according to the following: normal (N), benign with calcification (BC), benign with mass (BM), calcification (CALC) and mass (M).

**Figure 5:**
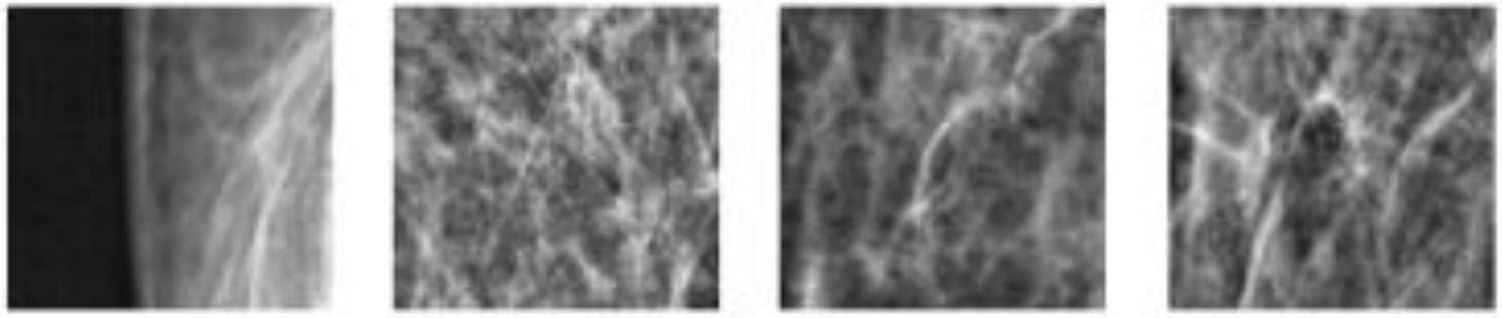
Four different samples with the normal (N) features of a healthy digital mammography.

**Figure 6:**
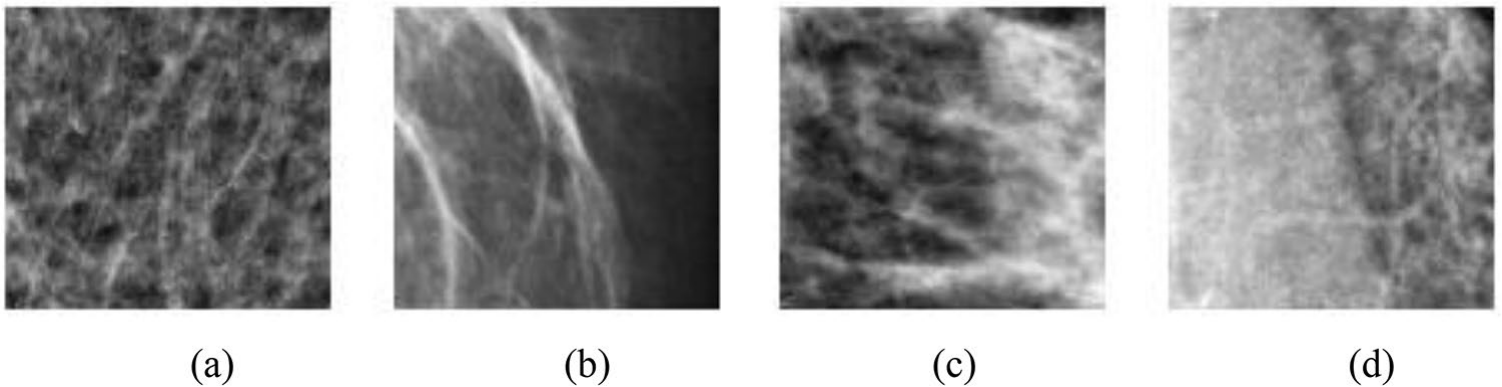
Samples of images to be extracted from a combined datasets sourced from DDSM + CBIS and MIAS databases. Image labels follows: (**a**) Mass abnormality (M), (**b**) calcification abnormality (CALC), (**c**) benign calcification (BC), and (**d**) benign with mass.

Figure 5 displays some digital mammography samples having normal representation of breast images. But in Fig. 6, we captured samples of the same modality having both benign and malignant abnormalities, and with characterization consistent with calcification and micro mass reported in the MIAS database.

**Figure 7:**
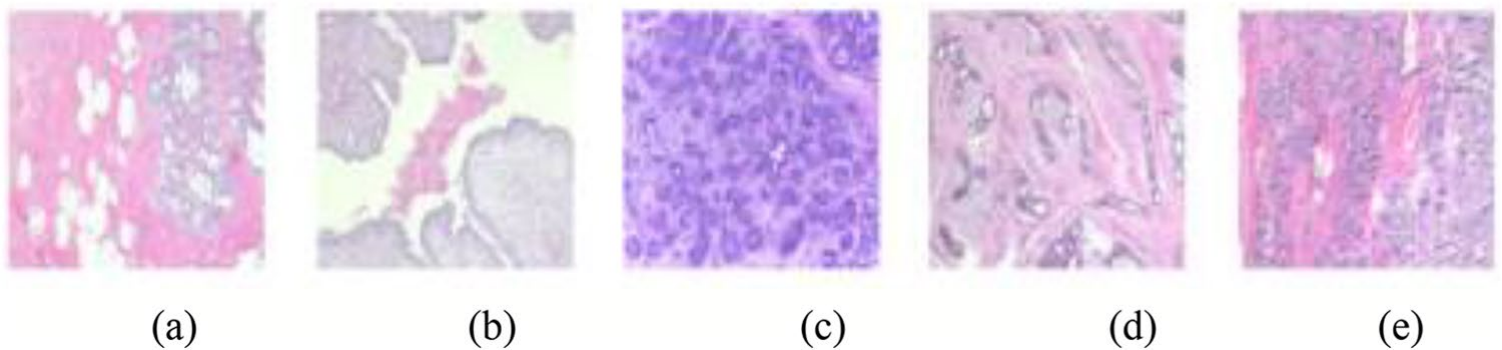
Samples drawn from a combination of BACH and BreakHis datasets showing benign abnormalities with (**a**) adenosis (A) from BreakHis, (**b**) phyllodes tumor (PT) from BreakHis, (**c**) benign (B) from BACH, (**d**) fibroadenoma (F) from BreakHis, and (**e**) tubular adenona (TA) from BreakHis.

**Figure 8:**
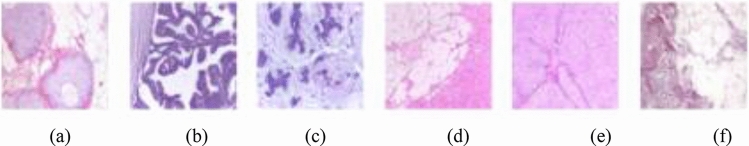
Samples drawn from a combination of BACH and BreakHis datasets showing malignant abnormalities with (**a**) carcinoma as malignant from BreakHis, (**b**) in situ carcinoma from BACH, (**c**) invasive carcinoma from BACH, (**d**) lobular carcinoma from BreakHis, (**e**) mucinous carcinoma from BreakHis, and (**f**) papillary carcinoma from BreakHis.

In Fig. 7, we show an array of samples with benign abnormalities and listing all the types of benign forms are represented in the BACH and BreakHis datasets. Similarly, Fig. 8 shows a listing of some selected samples having different types of malignant cases as reported in the BACH and BreakHis datasets.

Considering the difficulty of addressing multimodality problems due to challenging visual learning process, we applied transformational data augmentation technique to enrich and class-balance the datasets. Horizontal and vertical flips, and image angular rotation operations were applied to derive new samples to balance the image counts per class category. In total, mammoCNN architecture was trained using 107,346, while histoCNN was trained with 95,581 samples. The composition of histology datasets follows 51,511 original samples and 44,070 augmented samples, and while that of digital mammography comprises of 75,658 original samples and 31,688 augmented samples. The image data samples described here were applied for the full experimentation of the multimodal framework described in this study. In the next section, a detailed report on the results obtained is presented and discussed. Samples drawn from each modality were further resized to a 224 × 224 and 299 × 299 pixels for histology and mammography respectively. Furthermore, performance evaluation of the proposed framework is carried out for comparative analysis with state-of-the-art methods.

## Results and discussion

The results obtained for the complete experimentation are discussed in this section. First, the composing neural networks in TwinCNN are isolated and their performances investigated. This is to allow for understanding the suitability of the models in addressing the visual feature learning particular to the modality of input supplied. Furthermore, the study examined the effect on the binary optimization method supporting the histoCNN and mammoCNN retain discriminant features for an improved classification performance. Thirdly, and most importantly, the performance of the TwinCNN framework is studied and reported to demonstrate how the proposed fusion layer demonstrates suitability and good performance while solving the multimodal problem. The section is concluded by discussing the findings from the results obtained and outlying the contribution of the study.

### Performance of the histoCNN and mammoCNN on the features not optimized

**Figure 9:**
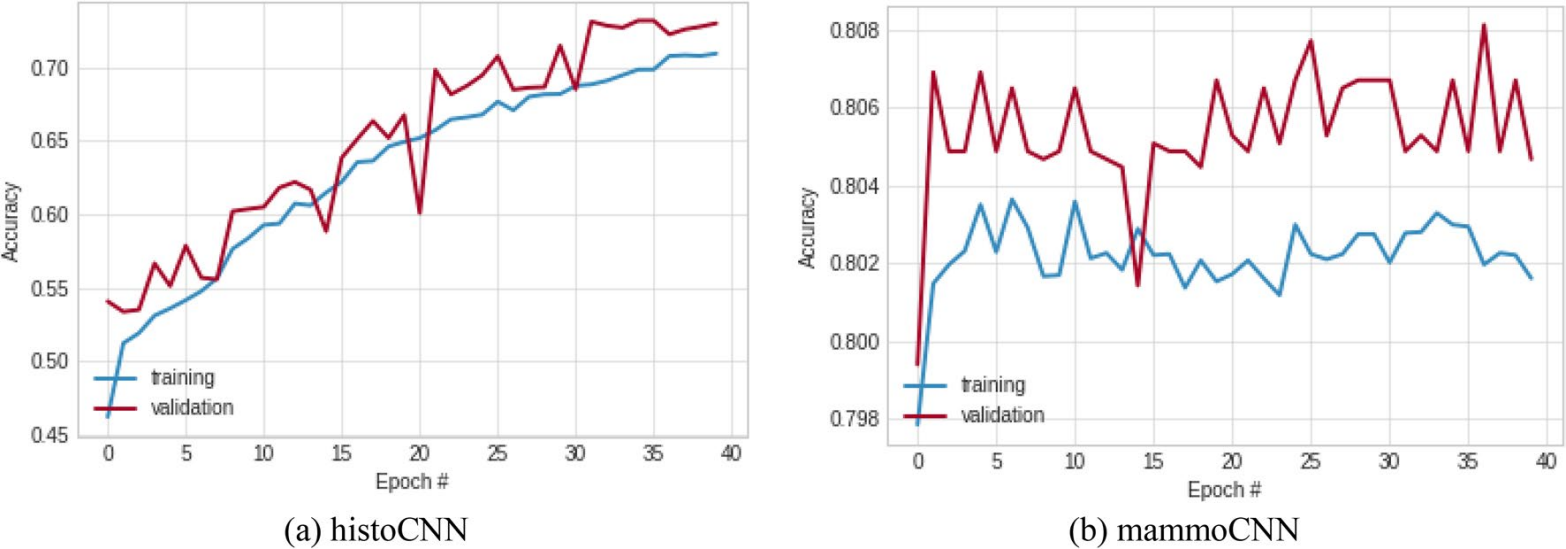
A plot showing the pattern of classification accuracy obtained for the training and validation of the (**a**) histoCNN and (**b**) mammoCNN when taken in isolation.

The histoCNN and mammoCNN models are understudied to investigate their suitability to function well in the TwinCNN architecture. This is necessary to ensure that the feature learning process adequately yields outputs which will contribute to the multimodal classification fusion output. In Fig. 9, the history classification accuracies for training and validation of histoCNN and mammoCNN over 40 epochs are plotted in two graphs. The observation made on the histoCNN model showed that the classification accuracy is significant both for training and validation with the highest values obtained in both cases are 0.709 and 0.729 respectively. Similarly, the performance of mammoCNN based on the classification accuracy of the training and validation were observed. Results obtained were plotted and they showed that only a slight difference exist between the training and validation result, with the value of 0.805 returned for the latter, while 0.802 for the former. This performance by the participating models of TwinCNN presents a motivation for addressing the multimodal problem.

**Figure 10:**
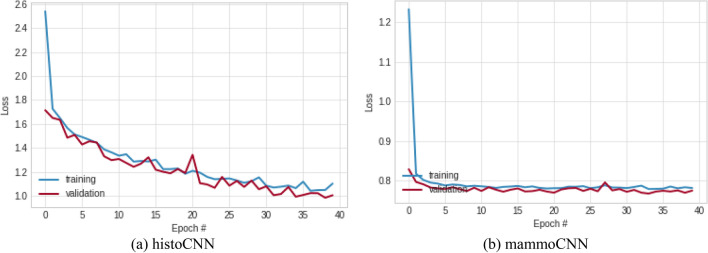
A plot showing the pattern of classification loss values obtained for the training and validation of the (**a**) histoCNN and (**b**) mammoCNN when taken in isolation.

The history of the loss function values was also monitored, and results collected for graphing as shown in Fig. 10. Performance demonstrating the learning pattern for histoCNN is impressive when the curves for training and validation are jointly considered. The best loss function values obtained for the histoCNN training and validation are 1.103 and 1.006 respectively. When these same best loss function values were observed for the mammoCNN model, the learning curves for training and validation also confirmed the suitability of the model for the multimodality task with the former yielding the lowest loss function of 0.781, and the latter returned 0.774.

Furthermore, to understand the suitability of the participating models in TwinCNN for achieving multimodal fusion task, we investigated the partially trained histoCNN and mammoCNN on different classifiers. Using the random forest (RF), K-Nearest Neighbor (KNN), multilayer perceptron (MLP) and the traditional Softmax function, this study evaluates the classification accuracy and area under curve (AUC) for the models. In Table 3, a summary of the results obtained are listed with the performance of the binary and multiclass classifiers compared and well performance classifier appears in bold font. The results obtained showed that all the classifier confirmed the suitability of models consisting of the TwinCNN with the MLP yielding the best classification accuracy of 0.952187 for histology modality while the RF yielded 0.799797 for mammography modality. In a similar vein, the MLP reported best AUc of 0.932702 while the RF has 0.673917 as the best. Meanwhile, those performance in terms of classification accuracy and AUC for other classifiers under the dual modalities are competitive. The average classification accuracy obtained for histology and mammography are 0.755325 and 0.791024 respectively, those for AUC 0.861871 and 0.637924 in that order. The implication of this performance evaluation is that the histoCNN and mammoCNN are very suitable and compactable for the TwinCNN operation. Moreover, further training of the models under higher epoch holds a promise of increasing the classification and accuracy and the AUC scores.

**Table 3:** Comparative analysis of the classification performance of the trained TwinCNN using the Softmax, KNN, RF, MLP and DTree algorithms.

Classifier	Histology		Mammography	
	Accuracy	AUC	Accuracy	AUC
KNN	0.788806	0.83349	0.780933	0.607248
RF	0.938817	0.917418	**0.799797**	**0.673917**
MLP	**0.952187**	**0.932702**	0.791684	0.632857
DTree	0.341491	0.763873	0.791684	0.637675
Softmax	0.708698	–	0.794726	–
Avg	**0.755325**	**0.861871**	**0.791024**	**0.637924**

**Figure 11:**
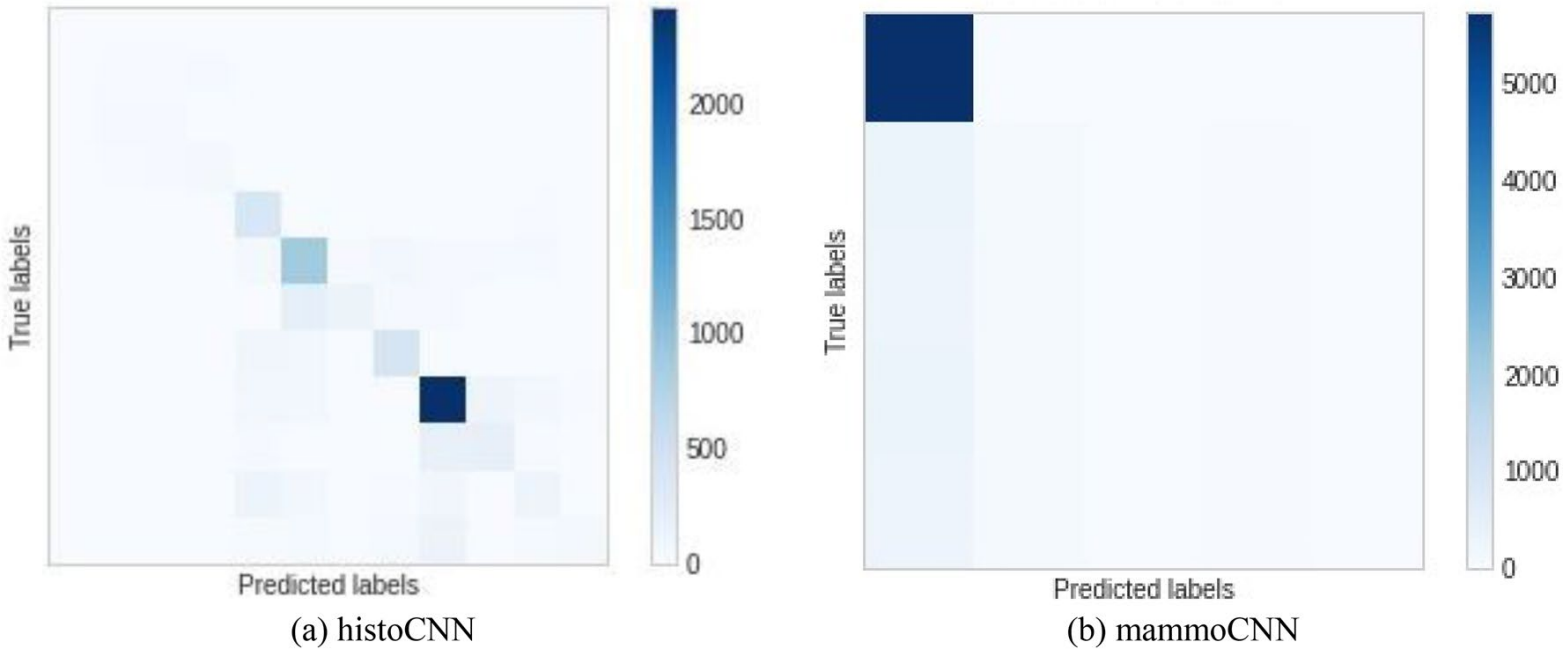
A confusion matrix plot for the (**a**) histoCNN using the learning rate of 0.001 and (**b**) mammoCNN when a learning rate of 1e−06 was applied for the experimentation.

Furthermore, Fig. 11 shows the confusion matrix obtained for the histoCNN and mammoCNN when applied for feature extraction and prediction in the TwinCNN framework. We found an interesting performance resulting from the histCNN, while the mammoCNN showed some difficulty in correctly learning features. As a result, we the model was further fine-tuned for better performance.

Considering the significant visual feature learning for the two modalities observed in this study, in the next subsection, the joint performance of the TwinCNN model is evaluated. Furthermore, the impact and performance output of the proposed fusion technique is also examined and reported.

### Performance of the TwinCNN with optimized features

The fused operation proposed in this study follows the non-traditional method which either fuses multimodal samples at neural architectural level or those that fuse at feature level. This subsection evaluates the adaptability of TwinCNN for fusion of the logits obtained from the individual model in the combined network. The approach reported here assumes that labels of the histology samples can be remapped from the original 12-label classes to a 3-label classes. Similarly, the mammography class labels were remapped from the 5-label classes to a 3-label classes. The remapping allows for standardization of the labels across all modalities for a fair fusion. Mammography label fusion resulted in = {“N”:[‘N’], “B”:[‘BC’, 'BM’], “M”:[‘CALC’, ‘M’]}, and the histology fusion resulted in {“N”:[‘N’], “B”:[ ‘B’, ‘A’, ‘F’, ‘PT’, ‘TA’], “M”:[‘IS’, ‘IV’, ‘DC’, ‘LC’, ‘MC’, ‘PC’]}. Note that the ‘N’, ‘B’, and ‘M’ denote the normal, benign, and malignant classes respectively. To allow for understanding the fusion process, we observed the probability values of the actual label and predicted labels for histology and mammography samples separately under their original 12 and 5 modal regimes respectively, and then carry out the fusion operation for further observation still under single modality. Finally, we applied multimodal fusion to understand what the final effect and strength of the classification is.

The results obtained are detailed and outlined in Table 4 where actual and predicted probability values for histology and mammography are listed. The first column lists the multimodal samples numbering. Here, randomly selected fused multimodal images of histology and mammography are used for the evaluation. The second and third columns are the values representing probability map for the true label and the label predicted by the histoCNN, while the fourth and fifth columns contains the probability values for the true labels and the predicted labels of randomly selected samples on mammoCNN. Under the category of columns titled fusion labels, the value for remapping the original 12-class regime for histology samples and the original 5-class regime for the mammography are listed in the first and second columns respectively. The values obtained for the class label remapping are foundational to the process of achieving multimodal fusion described in this study. Recall that to flatten their different labels counts representing different modalities, we need to find a collection of labels which can accurately represent all modalities. Hence the need to use the ‘N’, ‘B’, and ‘M’ labels. For the histology label remapping probabilities, we obtain 0.97702 for all randomly selected samples. For the mammography samples an interesting variation is reported for the randomly selected samples though this variation is not significant but demonstrates a similitude of what is obtainable with real samples.

**Table 4:** Comparing the performance of the probability fusion method with the actual and predicted labels of histoCNN and mammoCNN in the TwinCNN of ten (10) randomly selected fused samples.

Multimodal sample #	Histology label		Mammography label		Fusion labels		
	Actual	Predicted	Actual	Predicted	Histology	Mammography	Fused
1	1.0	0.954039	1.0	0.826078	0.97702	0.913039	0.683539
2	1.0	0.954039	1.0	0.826073	0.97702	0.913037	0.683538
3	1.0	0.954039	1.0	0.811109	0.97702	0.905555	0.679797
4	1.0	0.954039	1.0	0.755488	0.97702	0.877744	0.665892
5	1.0	0.954039	1.0	0.671327	0.97702	0.835664	0.644851
6	1.0	0.954039	1.0	0.742093	0.97702	0.871046	0.662543
7	1.0	0.954039	1.0	0.793881	0.97702	0.89694	0.67549
8	1.0	0.954039	1.0	0.756792	0.97702	0.878396	0.666218
9	1.0	0.954039	1.0	0.751871	0.97702	0.875935	0.664987
10	1.0	0.954039	1.0	0.711373	0.97702	0.855687	0.654863
11	1.0	0.954039	1.0	0.803643	0.97702	0.901821	0.67793
12	1.0	0.954039	1.0	0.799792	0.97702	0.899896	0.676967
13	1.0	0.954039	1.0	0.65444	0.97702	0.82722	0.64063
14	1.0	0.954039	1.0	0.826077	0.97702	0.913038	0.683539
15	1.0	0.954039	1.0	0.822246	0.97702	0.911123	0.682581
16	1.0	0.954039	1.0	0.789946	0.97702	0.894973	0.674506
17	1.0	0.954039	1.0	0.826076	0.97702	0.913038	0.683539
18	1.0	0.954039	1.0	0.722376	0.97702	0.861188	0.657613
19	1.0	0.954039	1.0	0.567527	0.97702	0.783764	0.618901
20	1.0	0.954039	1.0	0.757711	0.97702	0.878855	0.683539
Avg	1.0	0.954039	1.0	0.826078	0.97702	0.913039	0.667219

The last column in the category of the section labeled fusion labels, we have the probability values for the multimodal fusion reported. Here the values fuse the probability of the remapped histology and that of the remapped mammography for the randomly selected samples. An interesting result is obtained with most showing that probability values range between 0.60 and 0.68 and an average computed for these randomly selected samples is 0.667219.

**Figure 12:**
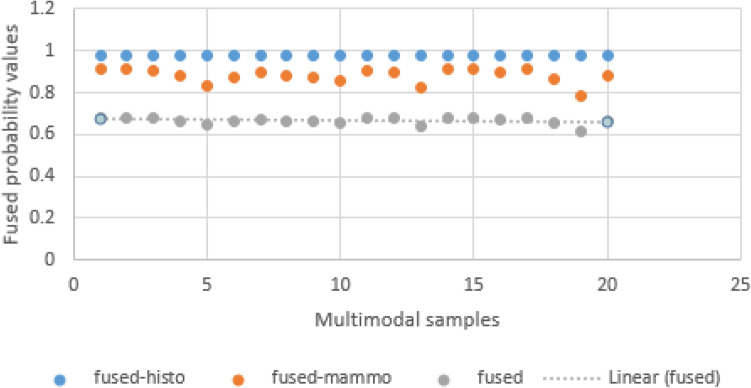
An illustration of the distribution of fused probability map values for 20 randomly selected single images with multimodal representation.

In Fig. 12, the probability values obtained for the remapped histology samples, mammography samples and the multimodal fusion are graphed to visualize any significant differences. The curves drawn on the graph show that all samples have values that fall within range with no weird point noticed. Again, this is important to under study the consistency of the mapping and fusion operations.

The aim of the fusion is to be able to jointly look at the result of different modalities and take an informed decision in deciding what the result to report to patients is—if a diagnosis is normal, benign, or malignant. In Table 5, we show that all randomly selected samples of histology were malignant, and the prediction also correctly labeled them as malignant. Similarly, original labels for the mammography samples selected for the evaluation were originally normal with the prediction labeling them as normal. Furthermore, we explored the labeling of the remapping. Interestingly, we noticed that the remapping models used also showed a consistency in the cases of histology and mammography samples correspondingly to those of actual and predicted. Now the most important stage in the pipeline is the fusion label. The ‘M–N’ label is displayed with a corresponding probability value to confirm the justification for the new label obtained.

**Table 5:** Comparing the performance of the class-based probability map fusion method with the actual and predicted labels of hisoCNN and mammoCNN in the TwinCNN of ten (10) randomly selected fused samples.

Multimodal sample #	Histology Label		Mammography label		Fusion labels			Probability of fused label
	Actual	Predicted	Actual	Predicted	Histology	Mammography	Fused	
1	M	M	N	N	M	N	M–N	0.683539
2	M	M	N	N	M	N	M–N	0.683538
3	M	M	N	N	M	N	M–N	0.679797
4	M	M	N	N	M	N	M–N	0.665892
5	M	M	N	N	M	N	M–N	0.644851
6	M	M	N	N	M	N	M–N	0.662543
7	M	M	N	N	M	N	M–N	0.67549
8	M	M	N	N	M	N	M–N	0.666218
9	M	M	N	N	M	N	M–N	0.664987
10	M	M	N	N	M	N	M–N	0.654863
11	M	M	N	N	M	N	M–N	0.67793
12	M	M	N	N	M	N	M–N	0.676967
13	M	M	N	N	M	N	M–N	0.64063
14	M	M	N	N	M	N	M–N	0.683539
15	M	M	N	N	M	N	M–N	0.682581
16	M	M	N	N	M	N	M–N	0.674506
17	M	M	N	N	M	N	M–N	0.683539
18	M	M	N	N	M	N	M–N	0.657613
19	M	M	N	N	M	N	M–N	0.618901
20	M	M	N	N	M	N	M–N	0.666447

The ‘M–N’ label is a fusion label describing the high possibility of the presence of malignancy in the reported samples and some form of normal classification also. However, the corresponding probability values justifies that the malignancy is more dominant than the normal label, thereby helping to explain the fusion label.

### Discussion of findings

In this section, a closer examination of the findings from the study is conducted from two perspectives: statistical analysis and comparative analysis of performance of the proposed multimodal neural network with similar state-of-the-art methods.

#### Statistical analysis

To statistically investigate the outcome of the multimodal fusion achieved using our TwinCNN, a t-test analysis was carried out. In this case, the analysis is based on the two modalities namely histology and mammogram to examine what differences exist in their extracted and predicted features that were used for the fusion. Specifically, the independent two-sample t-test was used for the analysis. Meanwhile, the null hypothesis considered to be tested is that there is not any difference between the two features using alpha = 0.05. The aim is to show that if there is no difference, then the outcome of the TwinCNN fusion model is not relevant. However, where a difference exists, then it implies that outcome from the fusion of the two modalities as reported by our experimentation is very valid.

First, an F-test to check the equality of the two modalities was analyzed as reported on Table 6. Results obtained showed that F > F Critical one-tail, therefore leading to the rejection of the consideration that both modalities are equal. For instance, the value 2.27E + 28 > 2.168252 is significant, demonstrating a necessary need for multimodality fusion. Furthermore, a two-tail test inequality analysis on the two modalities revealed an important finding which led to the rejection of the null hypothesis. To justify this, result in Table 7 confirms that the conditions tStat <− tCritical two-tail and tStat > tCritical two-tail do not hold. Supporting this argument is − 2.093024 < − 12.57598067 < 2.093024 condition which is true. Moreover, the observed difference between the sample means 0.88039795 and 0.97702 shows that a significant difference exists between the features from the two modalities.

**Table 6:** F-test result.

F-Test two-sample for variances		
	Mammo	Histology
Mean	0.880398	0.97702
Variance	0.001181	5.19E−32
Observations	20	20
Df	19	19
F	2.27E+28	
P(F < = f) one-tail	1.9E−265	
F Critical one-tail	2.168252

**Table 7:** t-Test outcome.

t-Test: two-sample assuming unequal variances		
	Mammo	Histology
Mean	0.880398	0.97702
Variance	0.001181	5.19E−32
Observations	20	20
Hypothesized Mean Difference	0	
Df	19	
t Stat	− 12.576	
P(T < = t) one-tail	5.85E−11	
t Critical one-tail	1.729133	
P(T < = t) two-tail	1.17E−10	
t Critical two-tail	2.093024	

In summary, considering the result of the statistical analysis, the confirmation of significant difference between the multimodal features demonstrates that the result of the TwinCNN fusion framework is impressive and relevant. In the next sub section, the approach proposed in this study is then compared with other similar related studies.

#### Comparison with similar studies

The comparison of our study with the state-of-the-art is first focused on emphasizing the differentiator between our approach and those similar studies on either twin or Siamese neural networks. Secondly, using performance metrics, results obtained from this study I compared with results from similar studies.

In Table 8, recent twin and Siamese neural networks are compared with our method by highlighting the differentiator existing between the two corresponding approaches. The works of^40,41,44,45,78^ and^37^ are all Siamese neural networks. A major differentiator with our approach lies in the use of the binary optimization algorithm as a basis for reduction of extracted feature, and as well for finding discriminant features supportive of the classification process. We consider this to be very important to obtain good prediction which can support the fusion process which depends on the predicted label and the multimodality features. On the other hand, the work reported in^39^ is rather an ensemble of neural networks used to build triplet Siamese network. While ensemble method has reported good performance in literature, we note that this can result in a very staggering dimension of features which needs to be considered during multimodality fusion. Interestingly, our method leverages a dual neural network approach for effectively extraction of the needed multimodal features. Furthermore, a very related work is that of^42^ which uses a twin neural network as in the case of our TwinCNN. However, our study differs from, and surpasses this related work because the multimodality investigated using TwinCNN is not the same as the multimodality reported in their study. Our consideration of multimodality is typical of a real-life medical image analysis on multiple modalities when detecting breast cancer.

**Table 8:** Approach-based comparative analysis of the proposed method with some selected related studies.

Studies	Approach	Domain of application	Differentiator with proposed study
^78^	Siamese convolutional neural network architecture called CNN-Siam, was applied to learn the feature representation of drug pairs from multimodal data of drugs	Prediction of drug-to-drug interactions (DDIs) based on modalities of chemical substructures, drug targets and enzymes	The RAdam and LookAhead optimization algorithms were relied on for improving accuracy based on ffeatures learned using the CNN-Siam, whereas our proposed model leverages a metaheuristic-based algorithm to select discriminant features learned using TwinCNN
^41^	Siamese neural network (SNN) is proposed for classification purpose in conjunction with k-nearest neighbour (k-NN) model	Applied to classification of MRI images samples for brain tumor detection	The study aimed at reducing feature space using shallow neural network as against the CNN architecture. On the contrary, our proposed TwinCNN is based on deep neural network capable of extracting rich features while a novel binary optimizer is applied for the feature space reduction
^44^	Deep learning-based Siamese neural network is design with attention mechanism	Detection of abnormality in product data at manufacturing site	The attention mechanism supports their feature extraction, it however introduces a very high representation of feature space Moreover, training of the model on small dataset contradicts our approach which leverages sufficient dataset to ensure that features space represents a good generalization
^39^	Triplet Siamese CNN based on benchmark architectures	Few-shot learning for detection of COVID-19	Ensemble of benchmark neural architectures were composed to build a triplet Siamese network. However, our proposed model is based on a dual neural architecture
^45^	Siamese CNN (SCNN) with minimal supervised learning	Applied for content-based retinopathy fundus image retrieval	Our proposed model combines features with predicted label for fusion which determines the multimodal classification
^40^	Siamese neural network based to enable one-shot classification	Handcrafted features were used to initiate the extraction of discriminant features	The study we propose leverages of binary optimizer with TwinCNN for feature extraction and selection of discriminant features
^37^	Siamese neural network for single modality image pair with two time points	Applied to monitor progression of disease	Our proposed TwinCNN is aimed at multimodal images combining histology and mammography samples
^42^	Twin CNN for extraction of feature maps based a content-based retrieval	Used for retrieval of Optical Coherent Tomography (OCT) scans	The TwinCNN proposed in our study is aimed for multimodality image classification with a novel feature extraction and reduction algorithm

The performance-based comparison listed in Table 9 shows how the proposed method competes with similar studies using some classification related metrics. Whereas most studies focused on single modality whose classification accuracies peaked higher our results, we consider such performance gain as non-comparable with combining the accuracy of multimodal classification. Moreover, the difficulty of finding studies which have proposed multimodality neural networks on histology and mammography datasets makes it challenging to draw a parallel performance comparison. Most importantly, we consider the unavailability of studies using the same combination of modalities as considered in our study on detection of breast cancer as confirmation of novelty.

**Table 9:** Performance-based comparative analysis of the proposed method with single/multiple neural networks for multimodality in detection of breast cancer detection.

Studies	Approach	Modalities	Performance
^79^	Two 3D ResNet-50 were combined for multimodal feature extraction and fusion	High‑dimensional MRI features and clinical information	AUC = 0.827
^80^	Integration of residual block with inception block to form a single CNN architecture	B-mode ultrasound, elastic ultrasound, pure elastic ultrasound, and H-channel images	Classification accuracy rates of breast lump detection is 94.76%
^81^	A single CNN architecture on B-mode and SE-mode ultrasound image	B-mode and elastography ultrasound images	sensitivity of 100 ± 0.00% and specificity of 94.28 ± 7.00%
^33^	A single neural architecture model for extracting stacked features using a sigmoid gated attention, and dense layer for bi-modality	Text-based, gene expression data and copy number alteration (CNA) data	Reported performance improvement for AUC, accuracy, precision, and sensitivity at 0.5%, 8.6%, 9.2% and 34.8% respectively
^82^	A single CNN architecture applied independently for extraction of multimodal features	Grey-scale images samples	Obtained 96.55%, 90.68%, and 91.28% on MIAS, DDSM, and INbreast datasets
This proposed study	A TwinCNN and binary optimization algorithm framework for multimodal classification using histology and mammography digital images	RGB-image and grey scale image samples	Classification accuracy for histology modality = 0.977, mammography modality = 0.913, and fused multimodalities = 0.684

Summary of results obtained in the study demonstrates that combining similar architectures for multimodal classification task is very important for good accuracy. Representing our approach as a twin network, we first evaluated the contributing models in TwinCNN and noted that each model supported the single-modality classification. Furthermore, the study demonstrates that inefficient models when combined as a twin architecture will impair the combined result of the hybrid neural architecture. Therefore, we motivate for studies on Siamese and Twin neural network to always examine their participating architectures individually and adjust or retrain them until they are suitable for participating in the joint twin/Siamese relationship. Secondly, the two models twined for fusion of samples from different modalities were adapted to remap their logits to a 3-class regime. Findings from this showed that mismatch representation of class in twin or Siamese network will lead to an imbalance multimodal classification. Therefore, it is good to find a baseline categorization of labels which allows for all participating modalities to be evaluated under the same type and number of labels. Finally, findings from the study demonstrate that the logits of neural architecture are also very useful in achieving fusion of such models when being used in twin or Siamese network architectures. Traditionally, all studies in literature have only limited their fusion levels to the architectural layers and the feature fusion levels. But this study, as far as we know, is the first attempt to investigate and experimentally show that the logits of twined or Siamese neural networks can also help in fusion of multimodal samples for a single classification report. This fusion probability is significant to whole pipeline of achieving a TwinCNN multimodal classification of breast cancer in images.

## Conclusion

This study is aimed at applying a novel TwinCNN framework to the task of extracting relevant heterogeneous patterns from multimodal datasets with the aim of addressing the difficult problem of multimodal image classification. We proposed an interesting technique for embedding binary optimization method to solve the problem of dimensionality reduction on the expected high volume of features extracted using the deep learning approach. Most studies which have addressed the challenge of feature optimization using metaheuristic algorithms have focused their methods on the application of continuous optimization algorithm. This study approached this problem from a different perspective using binary optimization algorithm. It represents a new direction from the popular method. It also motivates for a novel way to formalize the features as representatives of 1’s and 0’s only so that discriminant features are represented by 1’s while the non-discriminant are denoted by 0. Experimental results confirm the suitability of the approach proposed. Furthermore, the study presented a new fusion method contrary to the popular and deficient ones which are obtained in the literature. This framework addresses the issue of effectively extracting a common feature representation space from fused heterogeneous features. The TwinCNN architectures allowed for obtaining discriminant features from multimodal samples, and to further fuse the features based on the class distribution prediction. However, this detection and harnessing of discriminant features came at the computational cost of a binary optimizer algorithm. In addition, the approach proposed helps to eliminate the challenge of single supervised deep learning models which often rely on large datasets for training. Findings from the study showed that the classification accuracy of the multimodal method competes with state-of-the-art unimodal deep learning method. Secondly, the study also demonstrated that the combination different data streams to understanding the representation of a disease support the decision process and improves explainability of the performance of deep learning models solving medical image analysis. This is necessary considering the role of artificial intelligence in characterization of abnormalities in medical images. In future, recommend that research effort be directed towards investigating the integration of explanation facility which draws input from the learned features sets to provide evidence for the result obtained from the TwinCNN framework. Finally, the increasing use of attention mechanism in both visual and textual neural networks has gained research focus. We consider the possibility of integrating an attention mechanism into TwinCNN to make it more efficient.

## Data Availability

The datasets generated and/or analysed during the current study are available in the MIAS and BreakHis repositories https://wiki.cancerimagingarchive.net/pages/viewpage.action?pageId=22516629 and https://web.inf.ufpr.br/vri/databases/breast-cancer-histopathological-database-breakhis/.
